# Effects of Channels and Micropores in Honeycomb Scaffolds on the Reconstruction of Segmental Bone Defects

**DOI:** 10.3389/fbioe.2022.825831

**Published:** 2022-03-18

**Authors:** Keigo Shibahara, Koichiro Hayashi, Yasuharu Nakashima, Kunio Ishikawa

**Affiliations:** ^1^ Department of Biomaterials Faculty of Dental Science, Kyushu University, Fukuoka, Japan; ^2^ Department of Orthopedic Surgery, Graduate School of Medical Sciences, Kyushu University, Fukuoka, Japan

**Keywords:** honeycomb, scaffold, pore architecture, carbonate apatite, bone reconstruction

## Abstract

The reconstruction of critical-sized segmental bone defects is a key challenge in orthopedics because of its intractability despite technological advancements. To overcome this challenge, scaffolds that promote rapid bone ingrowth and subsequent bone replacement are necessary. In this study, we fabricated three types of carbonate apatite honeycomb (HC) scaffolds with uniaxial channels bridging the stumps of a host bone. These HC scaffolds possessed different channel and micropore volumes. The HC scaffolds were implanted into the defects of rabbit ulnar shafts to evaluate the effects of channels and micropores on bone reconstruction. Four weeks postoperatively, the HC scaffolds with a larger channel volume promoted bone ingrowth compared to that with a larger micropore volume. In contrast, 12 weeks postoperatively, the HC scaffolds with a larger volume of the micropores rather than the channels promoted the scaffold resorption by osteoclasts and bone formation. Thus, the channels affected bone ingrowth in the early stage, and micropores affected scaffold resorption and bone formation in the middle stage. Furthermore, 12 weeks postoperatively, the HC scaffolds with large volumes of both channels and micropores formed a significantly larger amount of new bone than that attained using HC scaffolds with either large volume of channels or micropores, thereby bridging the host bone stumps. The findings of this study provide guidance for designing the pore structure of scaffolds.

## Introduction

Despite technological advancements, segmental bone defects (SBDs) are intractable, and their suitable treatment scheme remains to be established (Bezstarosti et al., 2021; Norris et al., 2021). Frequent failure of SBD treatment may lead to functional disorders or amputations (Lasanianos et al., 2010; Mauffrey et al., 2015). In addition, the presence of SBDs leads to the loss of bone continuity, which increases risk of nonunion and renders the treatment difficult (Lasanianos et al., 2010; Mauffrey et al., 2015; Bezstarosti et al., 2021; Norris et al., 2021). Notably, critical-sized SBDs disrupt the bone orientation, resulting in a high rate of nonunion (Petersen et al., 2018). To achieve favorable outcomes in SBD treatments, a promising approach is to implant osteoconductive scaffolds that connect the stumps of the host bones and allow the penetration of various cells engaged in bone regeneration into the SBDs (Feng et al., 2012; Tanaka et al., 2017; Petersen et al., 2018).

Considering the aforementioned approach, we have been developing osteoconductive bioceramic honeycomb (HC) scaffolds with numerous channels directionally penetrating the scaffold (Hayashi et al., 2019a, 2019b, 2020b, 2020c, 2021c, 2021d, 2021e, 2022; Hayashi and Ishikawa 2020a, 2021a, 2021b; Sakemi et al., 2021; Shibahara et al., 2021). We confirmed that the presence of uniaxial channels in the HC scaffolds could promote bone ingrowth from the stumps of the host bone in SBD treatments (Sakemi et al., 2021; Shibahara et al., 2021). The HC scaffolds could realize adhesion to the bone and bone ingrowth as early or earlier than various three-dimensional porous scaffolds, and even combined three-dimensional porous scaffolds and mesenchymal stem cells (MSCs) (Zhou et al., 2010; Alluri et al., 2019). Other researchers also reported scaffolds that connected the stumps of the host bones without osteogenic growth factors, such as hydrogen-mineral composite (Patel et al., 2020), polylactide-coglycolide/tricalcium phosphate composite (Yu et al., 2018), and porous magnesium alloy (Wang et al., 2020). The ideal scaffold must be replaced with a new bone, while the scaffold parts corresponding to the bone marrow regions are resorbed (Zhang et al., 2020). However, previously developed HC scaffold did not achieve these requirements (Shibahara et al., 2021).

To surpass the efficacy of previous HC scaffolds and satisfy these requirements, an effective approach is to control the chemical composition and pore structure of the HC scaffold, which can crucially affect its osteoconductivity and resorption. In terms of the chemical composition, carbonate apatite (CAp) is resorbed by the osteoclasts at a similar pace as that of new bone formation and can thus be replaced with new bone (Doi et al., 1998; Zhang et al., 2021). In contrast, hydroxyapatite (HAp) is not resorbed and beta-tricalcium phosphate (β-TCP) is resorbed prior to new bone formation (Ishikawa et al., 2018; Hayashi et al., 2019b, 2020b). Considering the above properties of these calcium phosphate materials, CAp is desirable for the chemical composition of HC scaffolds in SBD reconstruction.

Existing studies on intrabony defect reconstruction and vertical bone augmentation demonstrated that HC scaffolds having channels with an aperture size of 190–300 μm can realize the ingrowth of bone and blood vessels more effectively than those with aperture sizes of 100–190 and 300–630 μm (Hayashi et al., 2020c, 2021d, 2021e, 2022; Hayashi and Ishikawa 2021a). Furthermore, the micropores affect osteoclastogenesis and the subsequent resorption of scaffolds by osteoclasts and new bone formation (Hayashi et al., 2019a; Hayashi and Ishikawa, 2020a). The scaffolds with both channels and micropores can exert osteoinduction and osteoconduction, and can shorten the bone reconstruction period (Woodard et al., 2007; Polak et al., 2011; Pei et al., 2017; Hayashi et al., 2020b). Thus, HC scaffolds with channels and micropores of suitable sizes are expected to exert superior efficacy for critical-sized SBD reconstruction.

Previously, researchers reported that three-dimensional scaffolds with optimized multiscale pores successfully reconstructed critical-sized SBDs in combination with osteogenic growth factors (Tang et al., 2016; Liu et al., 2019; Niu et al., 2019). However, these scaffolds were unable to reconstruct the critical-sized SBDs in the absence of osteogenic growth factors (Tang et al., 2016; Liu et al., 2019; Niu et al., 2019). This issue might be attributed to the focus on pore size only, while the importance of pore volume has been overlooked. Thus, the effects of pore volume on the SBD reconstruction efficacy remain unknown.

In this study, we investigated the efficacy of critical-sized SBD reconstruction using CAp HC scaffolds with different volume proportions of the channels and micropores to clarify the interrelation between these properties. Furthermore, we evaluated the SBD reconstruction efficacy of CAp HC scaffolds with suitable channel and micropore volumes.

## Materials and Methods

### Preparation of CAp HC Scaffolds

CAp HC scaffolds were fabricated by extrusion molding as described in our previous work (Hayashi and Ishikawa 2021a). In detail, a mixture of CaCO_3_ (Sakai Chemical Industry Co., Ltd., Osaka, Japan) and organic binder (Nagamine Manufacturing Co., Ltd., Kagawa, Japan) was extruded through the HC dies of a uniaxial extruder (Universe Co., Ltd., Saga, Japan). The organics in the HC green bodies were removed by sintering at 600–650°C in a CO_2_ atmosphere. Subsequently, CaCO_3_ HC blocks were obtained. The CaCO_3_ HC blocks were immersed in 1 mol/L Na_2_HPO_4_ (Fujifilm Wako Pure Chemical Co., Ltd., Osaka, Japan) at 80°C for 7 days. The chemical composition of the HC blocks was modified by transforming CaCO_3_ to CAp through dissolution–precipitation reactions to maintain the HC architecture. The prepared CAp HC blocks were washed at least five times with distilled water and shaped into cuboids (height, width, and length of 6, 3, and 10 mm, respectively) using computer-aided design and manufacturing tools (monoFab SPM-20, Roland DG, Shizuoka, Japan). Hereafter, the CAp HC scaffolds with a large channel volume and small micropore volume; small channel volume and large micropore volume; and large channel and micropore volumes are labeled c-HC, m-HC, and cm-HC, respectively.

### Characterizations of the CAp HC Scaffolds

The macro-/microstructures of the CAp HC scaffolds were examined through computer tomography imaging (µ-CT; Skyscan 1076, Bruker Co., Ltd., MA, United States) and scanning electron microscopy (S3400N, Hitachi High-Technologies Corporation, Tokyo, Japan). The crystal structure of the CaCO_3_ blocks and CAp HC scaffolds were determined through X-ray diffraction (XRD) analysis. The XRD patterns were recorded on a diffractometer (D8 Advance, Bruker AXS GmbH, Karlsruhe, Germany) with Cu Kα radiation of 40 kV and 40 mA. The chemical composition of the CaCO_3_ blocks and CAp HC scaffolds was examined through Fourier transform infrared spectroscopy (FTIR). The FTIR spectra were recorded on a spectrometer (FT/IR-6200; JASCO, Tokyo, Japan) using the KBr disk method. The standard XRD patterns and FTIR spectrum of HAp powder (HAP-100, Taihei Chemical Industries, Co., Ltd., Nara, Japan) were derived. Moreover, carbon–hydrogen–nitrogen (CHN) analysis (MT-6, Yanako Analytical Instruments, Kyoto, Japan) was performed to measure the carbonate content in the CAp HC scaffolds. The average carbonate contents were calculated from the results of three samples for each scaffold type. Mercury injection porosimetry (AutoPore 9420, Shimadzu Corporation, Kyoto, Japan) was performed to determine the size distribution and volume of open pores in the CAp HC scaffolds. The theoretical density of hydroxyapatite (3.16 g/cm^3^) was used to calculate the porosities of CAp because the chemical composition of CAp varies based on the amount of CO_3_ (Gibson and Bonfield 2001). In this analysis, eight samples for each scaffold type were tested. Furthermore, the compressive strength of the CAp HC scaffolds was measured as the mechanical strength using a universal testing machine (Autograph AGS-J, Shimadzu, Kyoto, Japan) installed with a load cell with the maximum capacity of 5,000 N. The samples were compressed parallel to the channel at a crosshead speed of 1 mm/min, and the value when sample fracture occurred was recorded. The mechanical strength test was performed using eight samples for each scaffold type.

### Animal Surgery

The animal experiments in this study were approved by the Animal Care and Use Committee of Kyushu University (Approval no. A21-010-0). Rabbits aged 18 weeks and weighing 2.9–3.4 kg have been used for SBD models (Okada et al., 1999; Kokubo et al., 2003; Suga et al., 2004). Ulnar or radial SBD models were used in this study owing to their low management cost (Horner et al., 2010). Previously reported SBD models of 10–20 mm ulna until 12 weeks postoperatively were considered critical-sized (Shibahara et al., 2021). Thus, in this study, a 10-mm-length SBD model of the rabbit ulna was selected to evaluate the SBD reconstruction efficacy of HC scaffolds over a short observation period (Supplementary Figure S1).

The rabbits were bred in the animal center of Kyushu University. The animal experiments were conducted according to the procedure used in our previous report (Shibahara et al., 2021). General anesthesia was performed by intramuscular injection of ketamine (30 mg/kg, Daiichi Sankyo Co., Ltd, Tokyo, Japan) and xylazine (5.0 mg/kg, Elanco Japan, Co., Ltd, Tokyo, Japan). After shaving and disinfecting the region of interest with 10% povidone iodine (Meiji Seika Pharma Co., Ltd., Tokyo, Japan), local anesthesia with lidocaine (2%, 6.0 mg/kg, Dentsply Sirona Co., Ltd., Tokyo) was provided through subcutaneous injection into the forearm. The skin on the lateral forearm was incised with a blade (Akiyama Medical MFG. Co., Ltd., Tokyo, Japan) to expose the ulna. The midshaft of the ulna was osteotomized along the side of a 10-mm-long guide block with a bone saw (sagittal blade; Zimmer Biomet Co., Ltd., Tokyo, Japan) to generate critical-sized SBDs (length of 10 mm) in the ulna (Shibahara et al., 2021). The periosteum and interosseous membrane were removed together with 10-mm-long bone fragment (Shibahara et al., 2021). Despite this procedure, bones are formed on the opposite bone by the periosteal reaction, and the generated bones are likely to elicit the bone formation in the defect (Bodde et al., 2008). In previous studies (Elbackly et al., 2015), GORE-TEX® was placed between the radius and the ulna to prevent the migration of cells from the radius and to eliminate the effect of periosteal reaction. Based on this report by Elbackly et al., in our study, a 10-mm-long cell-shielding membrane composed of polyethylene terephthalate was placed between the radius and the ulna to eliminate the effects of periosteal reaction from the radial side (Shibahara et al., 2021). The ulna was fixed using a nonlocking stainless-steel plate with a thickness of 0.6 mm. The scaffold was implanted in the bone defect and fixed to the plate with a 4-0 silk surgical suture (MANI Co., Ltd., Tochigi, Japan). The abovementioned procedures were implemented on both forearms, and the scaffold type (c-HC, m-HC, and cm-HC) inserted was randomly selected. Finally, the fasciae and skin were sutured with a 4-0 nylon surgical suture (MANI Co., Ltd.). To prevent wound infection, gentamicin (4 mg/kg, Takata Pharma Co., Ltd., Saitama, Japan) was injected intraperitoneally. The surgical site was again disinfected with 10% povidone iodine (Meiji Seika Pharma Co., Ltd.). The forearms were not immobilized after surgery, and the rabbits were allowed to move freely in their cages. Four weeks and 12 weeks postoperatively, the rabbits were euthanized with an anesthetic overdose, and both forearms were harvested (*n* = 4 per group).

### Radiographic Evaluation

After sacrifice, the reconstruction images of the specimens were checked by radiographs (HA-60, HITEX Co., Ltd,, Osaka, Japan). The bone formation at the osteotomized region and scaffold resorption were evaluated from the radiographs.

### µ-CT Evaluation

After removing the nonlocking stainless-steel plates and screws, the specimens were scanned through μ-CT (Skyscan 1076; Bruker Co., Ltd., MA, United States). The images were reconstructed using the NRecon software (Skyscan). The reconstructed images were evaluated based on their grayscale intensity. In the two-dimensional µ-CT image, threshold ranges were selected for the HC scaffold and bones to create segmentation images, respectively. Subsequently, the obtained segmentation images were compared with the original image to check whether they reflected their morphology (Bouxsein et al., 2010). The µ-CT images were observed to examine the long axis of the scaffolds 4 and 12 weeks postoperatively. The relevant values were calculated using Eqs 1–4.

BV/TV (%) = (volume of new bone in the scaffold)/(total volume of the bone defect) × 100 (1)

BV/CMV (%) = (volume of new bone in the scaffold)/(total volume of channels and micropores) × 100 (2)

SV/TV (%) = (volume of the scaffold)/(total volume of the bone defect) × 100 (3)

The volume percentage of remaining scaffolds (%) = (volume of the scaffold)/(volume of the scaffold before implantation) × 100 (4)

### Histological evaluation

Hematoxylin-eosin (HE)-stained tissue sections were prepared for histological evaluation. The long-axis images were evaluated considering a single cross-section along the ulnar shaft. The areas of the bones and blood vessels and the number of osteoclasts were estimated from the HE-stained tissue sections using a BZ-X digital analyzer (Keyence Corporation, Osaka, Japan). The multinucleated cells on the scaffold or bone were determined to be osteoclasts (Araújo et al. 2015). The relevant values were calculated using Eqs 5–8:

BA/TA(%) =(total area of bones formed in the defect)/(total defect area) × 100 (5)

BA/CMA(%) =(total area of bones formed in the defect)/(total area of channels and micropores) × 100 (6)

BVA/TA(%) =(area of the blood vessels)/(total defect area) × 100 (7)

$$\mathrm{Number of osteoclasts}\left(\mathrm{cells}/mm^{2}\right)=\left(\mathrm{number of osteoclasts}\right)/\left(\mathrm{total defect area}\right)$$ (8)

### Statistical Analysis

Statistical analysis was performed through a one-way analysis of variance followed by Tukey’s test. *p*-value < 0.05 was considered statistically significant.

## Results

### Physicochemical, Structural, and Mechanical Properties of CAp HC Scaffolds

In the XRD patterns, there were no CaCO_3_ (calcite) diffractions observed, whereas diffractions corresponding to apatite crystals were detected in all scaffolds (Supplementary Figure S2A). According to the FTIR spectra, the doublet carbonate bands were detected in the ν3 regions of all scaffolds (Supplementary Figure S2B). Although the FTIR spectrum of HAp presented a hydroxyl band at 630 cm^−1^, the FTIR spectrum of the CAp HC scaffolds did not exhibit this band (Supplementary Figure S2B). These results indicated that the chemical compositions of all scaffolds were AB-type CAp (Fleet et al., 2004). The CHN analysis highlighted that all scaffolds had a carbonate content of 13.0–14.8%, indicating their similar chemical compositions.

**FIGURE 1:**
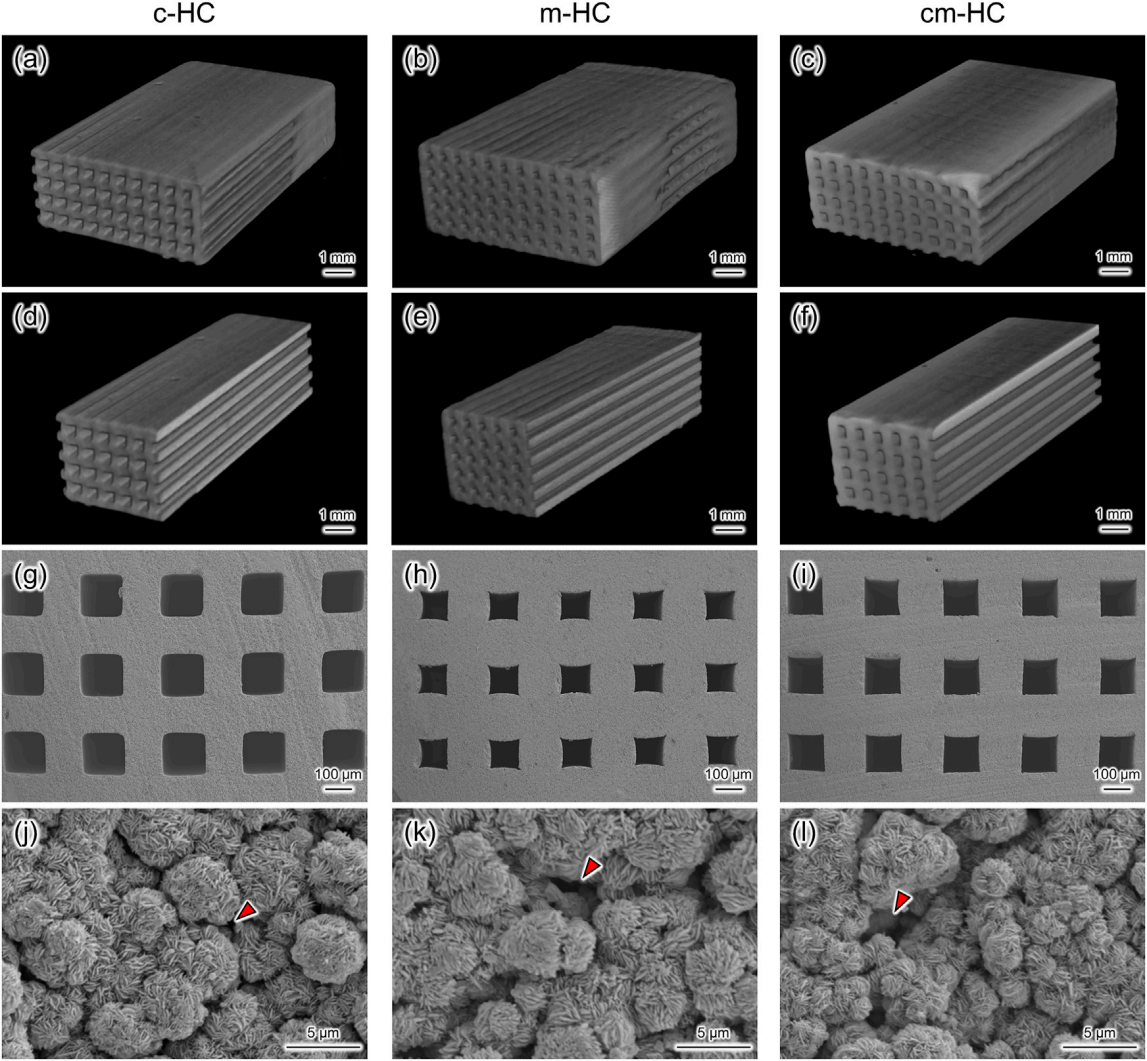
Three-dimensional µ-CT images of **(A)** c-HC, **(B)** m-HC, and **(C)** cm-HC. **(D–F)** Cross-sectional images of **(A–C)**, respectively. SEM images in the channel aperture region of **(G)** c-HC, **(H)** m-HC, and **(I)** cm-HC at low magnification. SEM images in the struts region of **(J)** c-HC, **(K)** m-HC, and **(L)** cm-HC at high magnification. Red arrowheads show the micropores formed in the struts of CAp HC scaffolds.

Uniformly sized channels uniaxially penetrated the scaffolds (Figures 1A–I). The channel aperture sizes of c-HC, m-HC, and cm-HC were 292.2 ± 4.7, 194.1 ± 6.8, and 259.1 ± 2.8 µm, respectively. In particular, the channel aperture sizes were suitable for the ingrowth of bone and blood vessels (190–300 µm) (Hayashi et al., 2020c, 2021d; Hayashi and Ishikawa 2021a). The sizes of the struts of c-HC, m-HC, and cm-HC were 256.8 ± 5.1, 320.7 ± 6.0, and 278.2 ± 4.0 µm, respectively. Micropores were formed in the struts of all scaffolds (Figures 1J–L).

**FIGURE 2:**
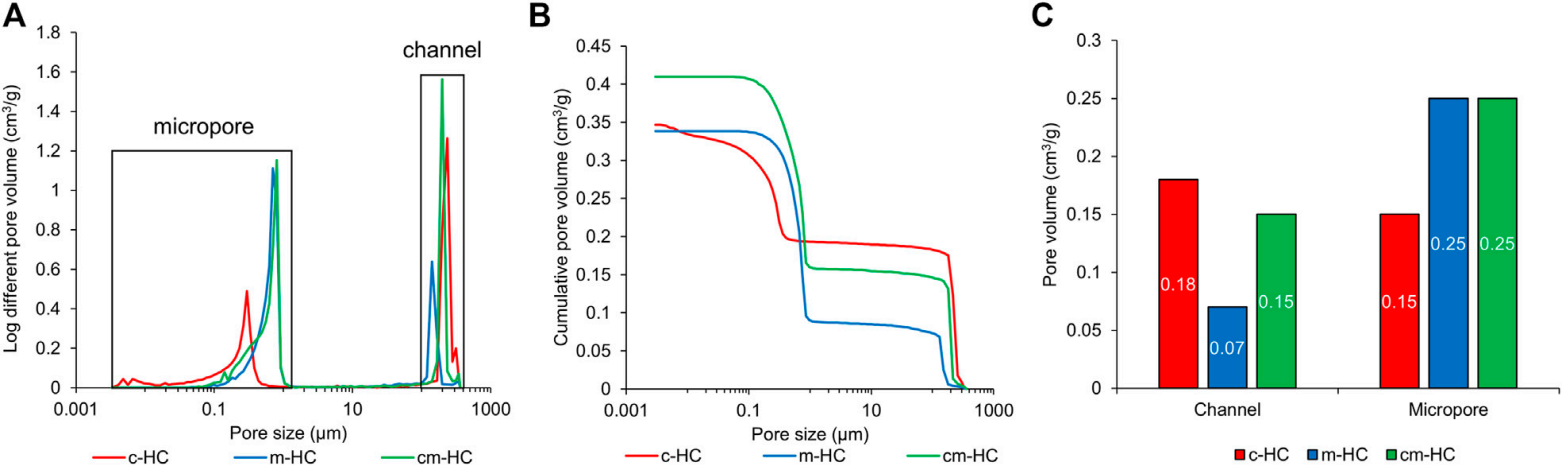
Results of mercury intrusion porosimetry for c-HC, m-HC, and cm-HC. **(A)** Pore-size distribution vs. pore diameter, **(B)** cumulative pore volume vs. pore diameter, and **(C)** pore volumes of channels and micropores.

The pore volume and distribution were measured through mercury intrusion porosimetry. All scaffolds possessed pores of sizes greater than 100 µm and less than 1 μm, corresponding to the channels and micropores, respectively (Figure 2A). The channel volumes in c-HC, m-HC, and cm-HC were 0.18, 0.07, and 0.15 cm^3^/g, respectively (Figures 2B,C). The micropore volumes in c-HC, m-HC, and cm-HC were 0.15, 0.25, and 0.25 cm^3^/g, respectively (Figures 2B,C). Thus, the combined volume of the channels and micropores in c-HC (0.33 cm^3^/g) was nearly equal to that in m-HC (0.32 cm^3^/g), although the volume ratio between the channels and micropores was different. The combined volume of the channels and micropores in cm-HC was 25% larger than those in c-HC and m-HC.

**FIGURE 3:**
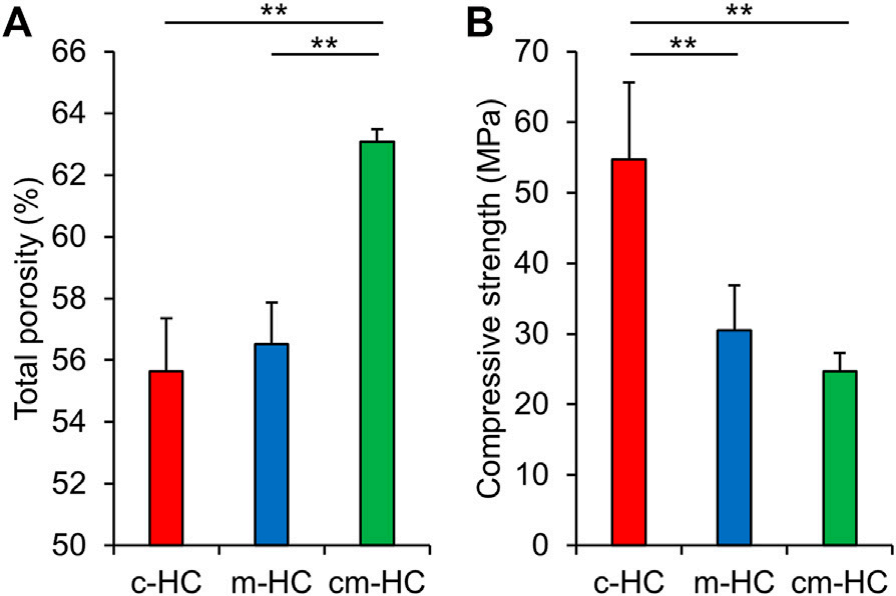
**(A)** Total porosities and **(B)** compressive strength of c-HC, m-HC, and cm-HC. *p < 0.05 and **p < 0.01.

The porosities of c-HC, m-HC, and cm-HC were 55.7 ± 1.7%, 56.5 ± 1.3%, and 63.1 ± 0.5%, respectively (Figure 3A). Thus, the porosity of c-HC was nearly equal to that of m-HC, whereas the porosity of cm-HC was significantly higher than those of c-HC and m-HC (*p* < 0.01), consistent with the results of the mercury intrusion porosimetry. The compressive strength values for c-HC, m-HC, and cm-HC were 54.7 ± 10.9, 30.4 ± 6.4, and 24.7 ± 2.6 MPa, respectively (Figure 3B). All scaffolds possessed a higher compressive strength than those of the scaffolds used for clinical treatment (Tanaka et al., 2008, 2018; Onodera et al., 2014). Furthermore, the compressive strength of c-HC was significantly higher than those of m-HC and cm-HC (*p* < 0.01), whereas those of m-HC and cm-HC were comparable. This finding indicated that the compressive strength was highly influenced by the presence of micropores in the struts than the channels.

### Radiographic Evaluation

**FIGURE 4:**
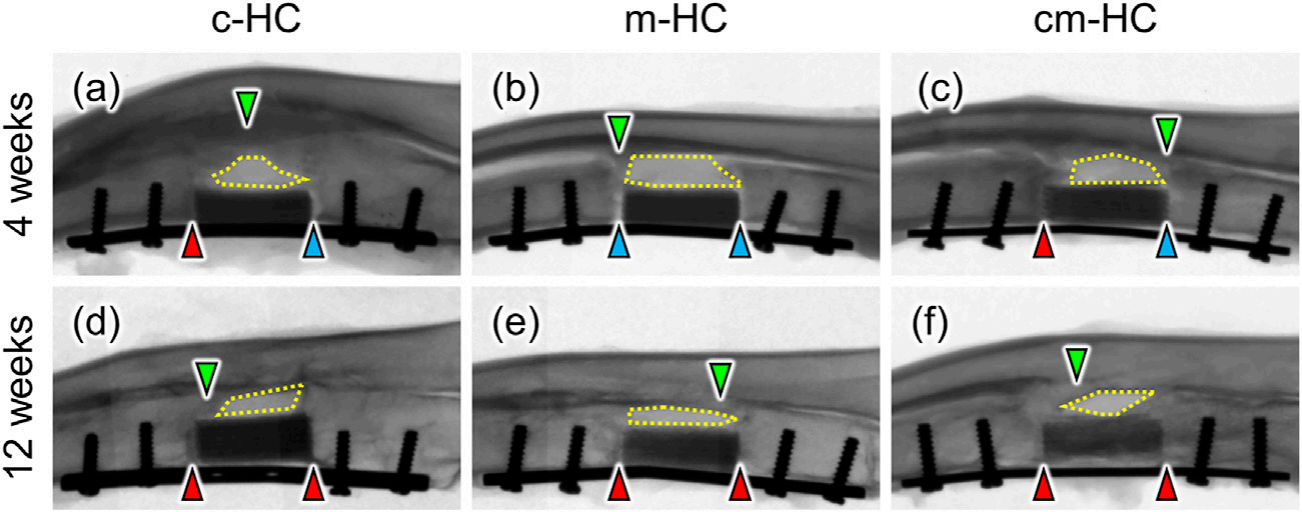
Typical radiographic images of **(A)** c-HC, **(B)** m-HC, and **(C)** cm-HC groups 4 weeks postoperatively and **(D)** c-HC, **(E)** m-HC, and **(F)** cm-HC groups 12 weeks postoperatively. The red arrowheads indicate osseointegration between the scaffold and host bones. The blue arrowheads indicate gaps between the scaffold and host bones. The green arrowheads indicate bone formation on the radius. The regions enclosed by yellow dotted lines are gaps between scaffolds and the radius. The left and right sides of the radiographs are the proximal ulna and the distal ulna, respectively.

Four weeks postoperatively, the proximal gap between the scaffold and the host bone was filled with new bones in c-HC and cm-HC, whereas both proximal and distal gaps were not observed in m-HC (Figures 4A–C). Twelve weeks postoperatively, both gaps were filled with new bones in all groups (Figures 4D–F). The intensity in c-HC did not vary visibly from four to 12 weeks postoperatively (Figures 4A,D), whereas that in m-HC and cm-HC decreased at 12 weeks postoperatively (Figures 4B,C,E,F), suggesting that m-HC and cm-HC were partially resorbed during 8 weeks. Four weeks and 12 weeks postoperatively, newly formed bones on the radius neither made contact with HC scaffolds nor got into the defects, indicating that scaffolds were not affected by the bones on the radius (Figures 4A–F).

### µ-CT Evaluation

**FIGURE 5:**
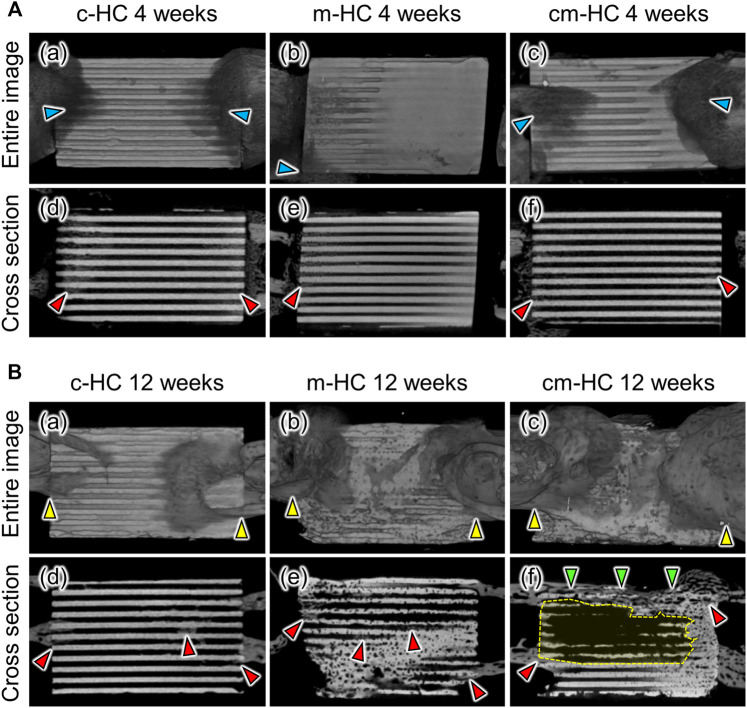
Typical µCT images of **(A,D)** c-HC, **(B,E)** m-HC, and **(C,F)** cm-HC groups **(A)** at 4 weeks postoperatively and **(B)** at 12 weeks postoperatively. The red, blue, yellow, and green arrowheads indicate new bone formation, bony calluses formed around the edge of scaffolds, osseointegration between the scaffold and host bones, and bony bridging in the defect, respectively. The regions enclosed by yellow dotted lines show that most of the channels were resorbed and began to be replaced by bone marrow.

Four weeks postoperatively, bony calluses were formed around the scaffolds (Figure 5Aa‒c), and new bone tissues emerged in the regions between the stump of the host bone and the scaffold (Figure 5Ad‒f). Notably, there was a significant formation of bony calluses and new bones for c-HC (Figure 5Aa,d). Clear scaffold resorption was not visualized, and the HC structure was maintained in all scaffolds.

Twelve weeks postoperatively, osseointegration was achieved in all groups (Figure 5Ba‒c). In particular, new bone formation remarkably covered both edges of cm-HC (Figure 5Bc). In the case of c-HC, although the struts were slightly resorbed, the HC structure was maintained (Figure 5Bd). New bone was also partially formed within the channels. In the case of m-HC, the struts were resorbed and became thinner than those at 4 weeks (Figure 5Be). New bone was widely formed in the interior of the scaffold. In the case of cm-HC, the new bones formed on the surface and inside the scaffold in the regions corresponding to the bone substance and consequently connected the separated host bones (Figure 5Bf). Furthermore, in the region corresponding to the bone marrow, the scaffold was extensively resorbed (Figure 5B-f). Thus, cm-HC was replaced with tissues similar to the actual bone in the SBD (Figure 5Bf).

**FIGURE 6:**
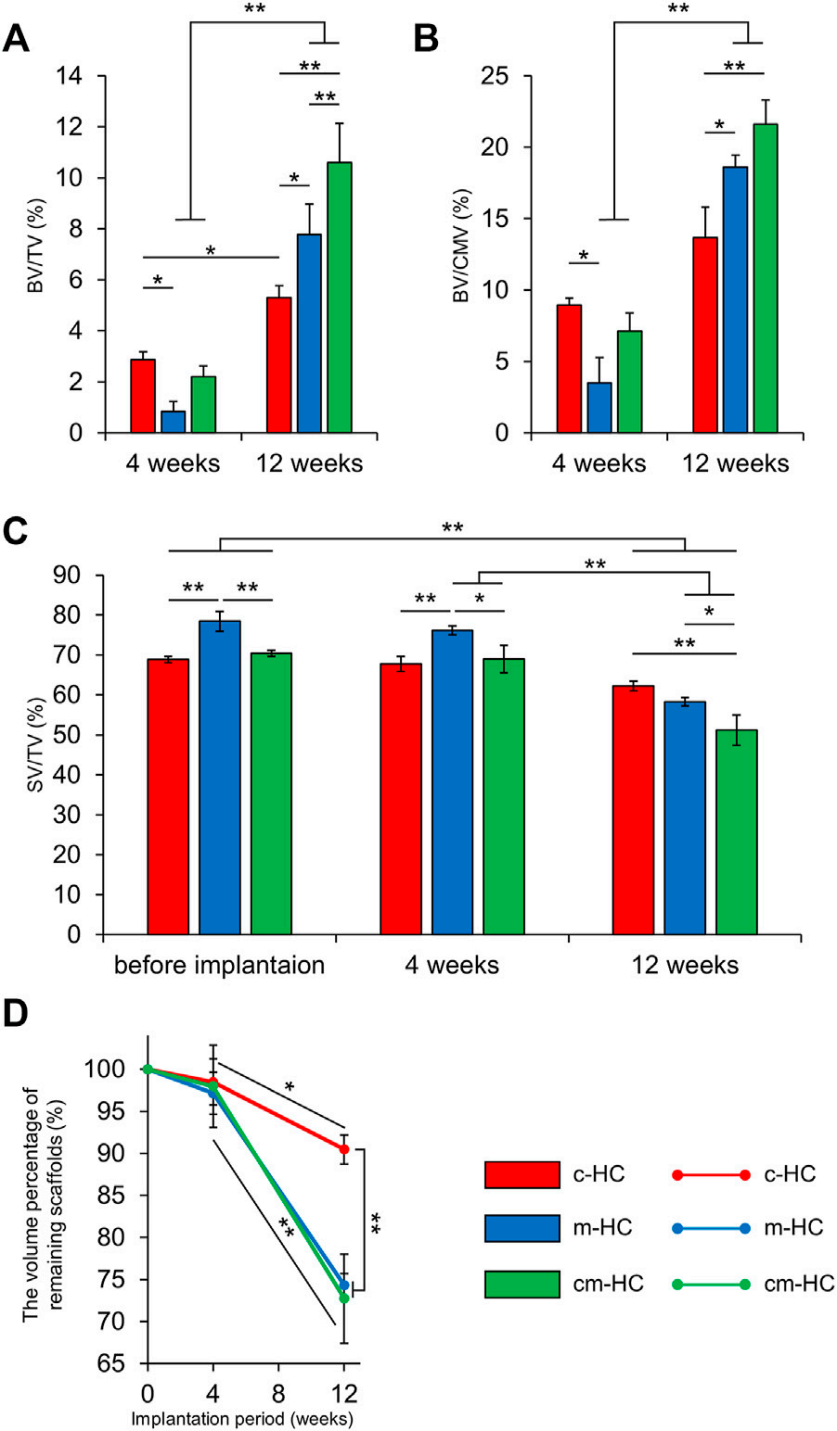
µ-CT analysis results. **(A)** BV/TV, **(B)** BV/CMV, **(C)** SV/TV, and **(D)** the volume percentage of remaining scaffolds. *p < 0.05 and **p < 0.01.

BV/TV (%), BV/CMV (%), SV/TV, and the volume percentage of the remaining scaffolds (%) were calculated by analyzing the µ-CT images (Figures 6A–D). The BV/TV values for c-HC, m-HC, and cm-HC were 2.9 ± 0.3%, 0.8 ± 0.4%, and 2.2 ± 0.4% 4 weeks postoperatively, and 5.3 ± 0.5%, 7.8 ± 1.2%, and 10.6 ± 1.5% 12 weeks postoperatively, respectively (Figure 6A). The BV/CMV values for c-HC, m-HC, and cm-HC were 8.9 ± 0.5%, 3.5 ± 1.8%, and 7.1 ± 1.3% 4 weeks postoperatively and 13.7 ± 2.1%, 18.6 ± 0.8%, and 21.6 ± 1.7% 12 weeks postoperatively, respectively (Figure 6B). SV/TV for c-HC, m-HC, and cm-HC were 68.8 ± 0.8%, 78.4 ± 2.5%, and 70.4 ± 0.8% before implantation, 67.8 ± 1.9%, 76.2 ± 1.1%, and 68.9 ± 3.5% 4 weeks postoperatively, and 62.2 ± 1.2%, 58.3 ± 1.1%, and 51.2 ± 3.7% 12 weeks postoperatively, respectively (Figure 6C). The volume percentages of the remaining scaffolds for c-HC, m-HC, and cm-HC were 98.5 ± 2.7%, 97.2 ± 2.5%, and 98.0 ± 4.9% 4 weeks postoperatively and 90.5 ± 1.7%, 74.3 ± 1.4%, and 72.7 ± 5.3% 12 weeks postoperatively, respectively (Figure 6D).

At 4 weeks postoperatively, the bone volume percentage in the c-HC group was higher than that in the m-HC group. There was no difference in the percentage of the scaffold resorption in all groups. At 12 weeks postoperatively, c-HC had the lowest percentage of scaffold resorption. The BV/CMV in c-HC showed no significant increase in 8 weeks, indicating the delayed bone formation relative to the scaffold resorption in the middle stage. Therefore, channels had a significant impact on early bone formation and poor impact on medium-term bone formation. The m-HC and cm-HC scaffolds showed significant increments of bone formation from four to 12 weeks postoperatively despite the significant scaffold resorption, indicating that the volume of the bones increased more than that of the scaffold resorption.

The findings indicated that the effect of the channels on bone formation was higher than that of the micropores 4 weeks postoperatively. The differences in the volumes of the channels and micropores did not affect the scaffold resorption. Twelve weeks postoperatively, the effect of the micropores on both scaffold resorption and bone formation was more notable than that of the channels.

### Histological Evaluations

**FIGURE 7:**
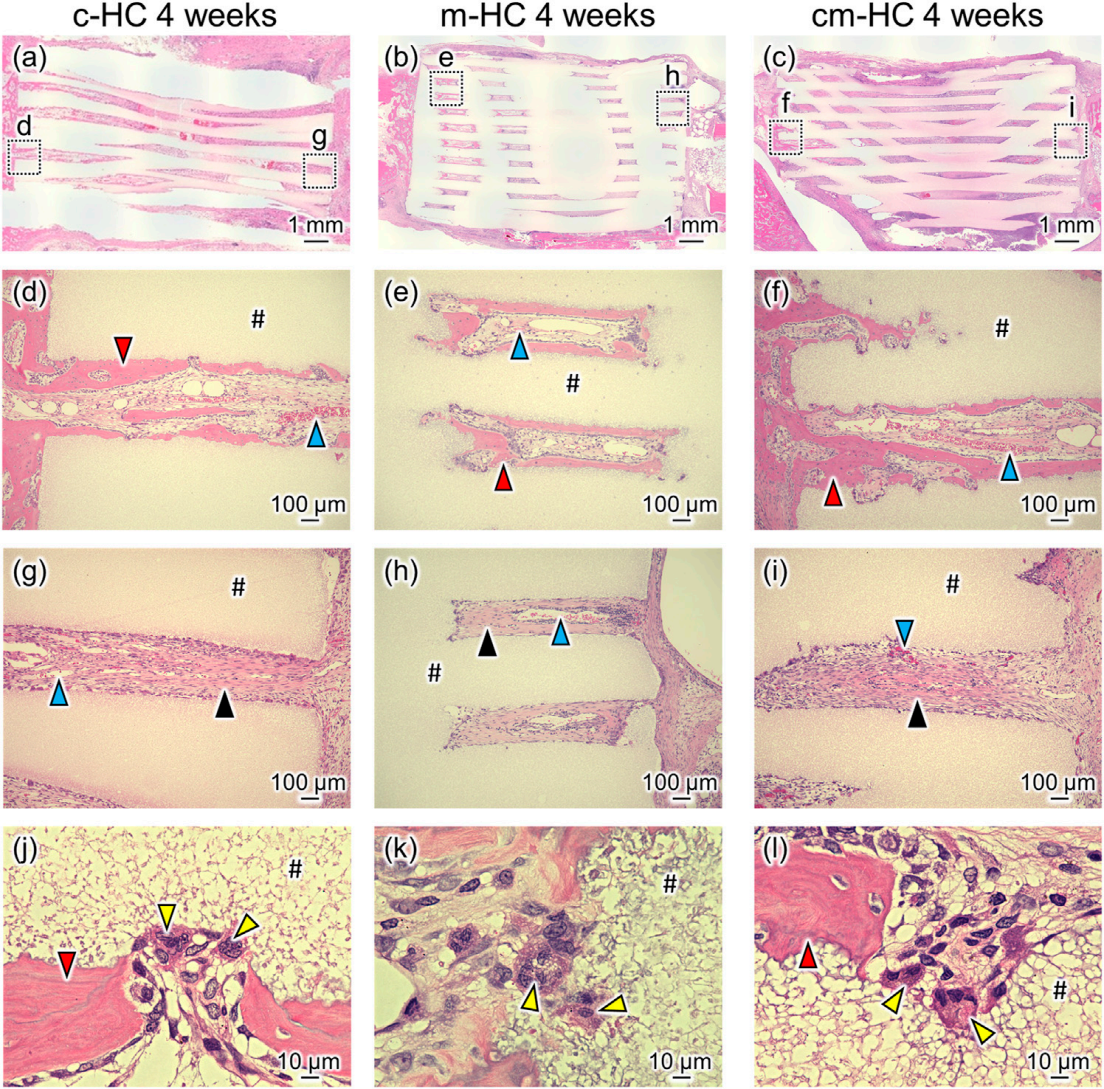
Typical HE-stained images **(A)** c-HC, **(B)** m-HC, and **(C)** cm-HC groups 4 weeks postoperatively. Postoperatively, **(D,G)**, **(E,H)**, and **(F,I)** correspond to the low-magnification images of the region enclosed by dotted lines in **(A–C)**, respectively; **(J–L)** are the high-magnification images of **(D–F)**, respectively. The red, blue, yellow, and black arrowheads indicate mature bone inside the channel, blood vessel, osteoclast, and fibrous tissue, respectively. “#” indicates the material.

Four weeks postoperatively, pronounced resorption of the scaffold was not observed for any scaffolds (Figures 7A–C), consistent with the µ-CT image findings. New bones formed on the strut surfaces and blood vessels were formed within channels contacting the host bone (Figures 7D–F). However, in the regions where the scaffolds did not contact the host bone, fibrous tissues were formed within channels (Figures 7G–I). In all groups, osteoclasts were present on the scaffold struts (Figures 7J–L).

**FIGURE 8:**
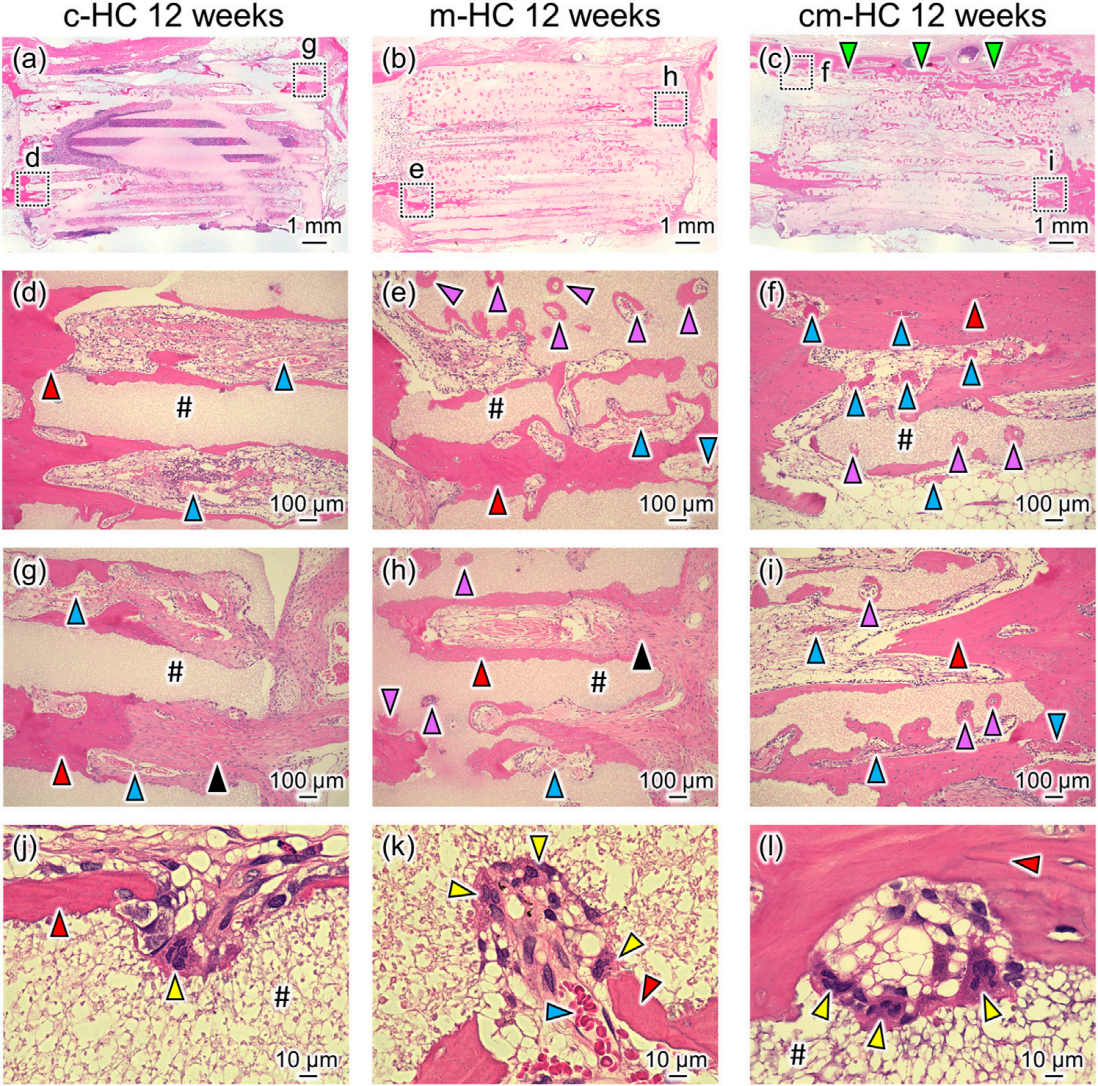
Typical HE-stained images **(A)** c-HC, **(B)** m-HC, and **(C)** cm-HC groups 12 weeks postoperatively. Postoperatively, **(D,G)**, **(E,H)**, and **(F,I)** correspond to the low-magnification images of the region enclosed by dotted lines in **(A–C)**, respectively; **(J–L)** are the high-magnification images of **(D–F)**, respectively. The red, purple, blue, yellow, and black arrowheads indicate mature bone within the channel, mature bone in the resorption lacunae formed in the struts, blood vessel, osteoclast, and fibrous tissue, respectively; “#” indicates the material. The green arrowhead indicates the bony bridging in the defect.

Twelve weeks postoperatively, m-HC and cm-HC were more extensively resorbed than c-HC (Figures 8A–C). In the case of c-HC, new bones were formed within the channels along the strut surface, whereas fibrous tissue was still observed within some channels at the aperture region (Figures 8D,G). In the case of m-HC, new bones were formed both on the strut surfaces and in the resorption lacunae formed in the struts (Figures 8E,H). However, as with c-HC, fibrous tissue was also observed within some channels at the aperture region (Figure 8H). The struts in cm-HC were more extensively replaced with new bones than those in m-HC. Few fibrous tissues were observed within channels at the aperture region (Figures 8F,I). Notably, bony bridging was observed between the host bones, and the scaffold corresponding to ulnar bone marrow was filled with bone marrow (Figure 8C and Supplementary Figure S3), suggesting bone remodeling in cm-HC. In terms of vascularization in the scaffold, well-developed blood vessels were observed in the edge region of the scaffolds, whereas in the central region of the scaffolds, thin blood vessels were observed within some channels at 4 weeks postoperatively (Supplementary Figure S4A). Twelve weeks postoperatively, well-developed blood vessels were observed in both scaffold edge and central regions, notably in the cm-HC (Supplementary Figure S4B). In all scaffolds, osteoclasts were present on the strut surfaces (Figures 8J–L).

**FIGURE 9:**
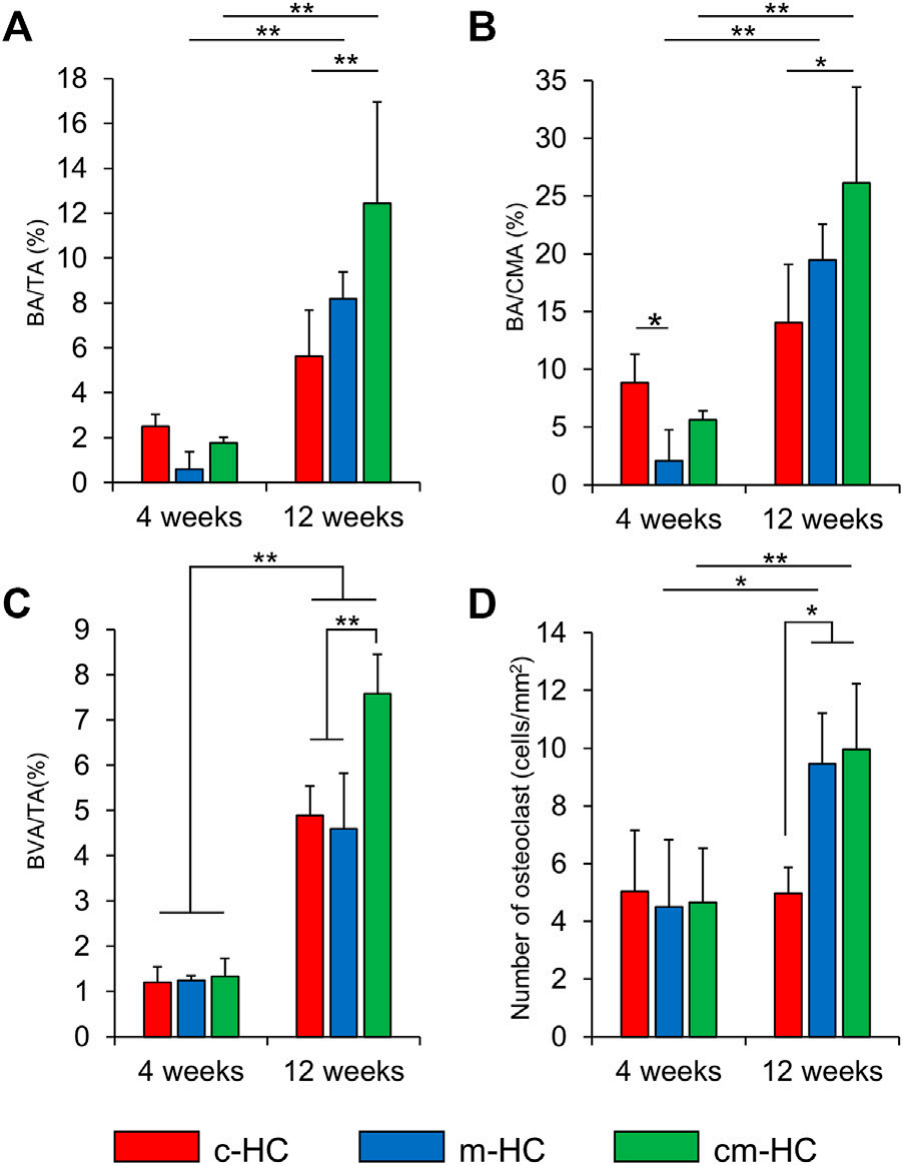
Histological analysis results. **(A)** BA/TA (%), **(B)** BA/CA (%), **(C)** BVA/TA (%), and **(D)** number of osteoclasts (cells/mm^2^); *p < 0.05 and **p < 0.01.

BA/TA, BA/CMA, BVA/TA, and the number of osteoclasts were calculated from the histological observations of the c-HC, m-HC, and cm-HC groups (Figures 9A–D). The BA/TA values for the c-HC, m-HC, and cm-HC groups were 2.5 ± 0.5%, 0.6 ± 0.8%, and 1.8 ± 0.2% 4 weeks postoperatively; and 5.6 ± 2.0%, 8.2 ± 1.2%, and 12.4 ± 4.5% 12 weeks postoperatively, respectively (Figure 9A). The BA/CMA values for the c-HC, m-HC, and cm-HC groups were 8.8 ± 2.4%, 2.1 ± 2.7%, and 5.6 ± 0.8% 4 weeks postoperatively; and 14.1 ± 5.0%, 19.5 ± 3.1%, and 26.2 ± 8.3% 12 weeks postoperatively, respectively (Figure 9B). BVA/TA for the c-HC, m-HC, and cm-HC groups were 1.2 ± 0.3%, 1.2 ± 0.1%, and 1.3 ± 0.4% 4 weeks postoperatively; and 4.9 ± 0.7%, 4.6 ± 1.2%, and 7.6 ± 0.9% 12 weeks postoperatively, respectively (Figure 9C). The numbers of osteoclasts for the c-HC, m-HC, and cm-HC groups were 5.0 ± 2.1, 4.5 ± 2.3, and 4.7 ± 1.9 cells/mm2 4 weeks postoperatively; and 5.0 ± 1.0, 9.5 ± 1.8, and 10.0 ± 2.3 cells/mm^2^ 12 weeks postoperatively, respectively (Figure 9D).

Four weeks postoperatively, the bone area percentage in the channels and micropores of c-HC was significantly larger than that of m-HC (*p* < 0.05), although there was no significant difference observed in the bone area percentage between c-HC and cm-HC. For all groups, the bone area percentages increased in 8 weeks. The increments in the m-HC and cm-HC groups were larger than that in the c-HC group. These results for the bone formation were highly consistent with that obtained from the µ-CT analysis. The semiquantification of the vascularization indicated that the area of blood vessels increased in all groups from 4 weeks and 12 weeks postoperatively, and the increase in cm-HC at 12 weeks postoperatively was the highest among all the groups (Figure 9C). The numbers of osteoclasts were equal for all groups after 4 weeks. Twelve weeks postoperatively, the numbers of osteoclasts in the m-HC and cm-HC groups were significantly larger than those at 4 weeks; the same phenomenon was not observed for the c-HC group. The abovementioned *in vivo* results demonstrated that the channels promoted the bone ingrowth in the early stage, and the micropores promoted the scaffold resorption, bone formation, and replacement of the HC scaffolds with new bone in the medium term.

## Discussion

The obtained findings demonstrated that the channels promoted bone ingrowth in the early stage, and the micropores promoted the scaffold resorption by osteoclasts, bone formation, and replacement of scaffolds with new bone in the medium term. Thus, the influence of the channels and micropores was different at different time points in the SBD reconstruction. In addition, when the scaffolds had both suitable channel and micropore volumes, they were successfully replaced with new bone. The roles of the channels and micropores in the HC scaffold for the biological responses in SBD reconstruction are discussed in detail below.

For the channel effects, we previously reported the ability of CAp HC scaffolds to reconstruct SBD with a channel aperture size of 146 μm, a channel volume of 0.16 cm^3^/g, and a micropore volume of 0.17 cm^3^/g using the same animal experiment as that in this study (Hayashi and Ishikawa 2021a; Shibahara et al., 2021). Thus, the channel aperture size of the CAp HC scaffolds used in our previous study was half that of c-HC in the present study, whereas these two HC scaffolds had nearly equal channel and micropore volumes. Importantly, the amount of the new bone was equal for these two HC scaffolds. The above results demonstrated that bone ingrowth in the early stage was affected by the channel volume rather than the channel size, when the channel size was suitable for promoting the bone ingrowth into the scaffold (>100 µm) (Karageorgiou and Kaplan 2005; Perez and Mestres 2016; Bobbert and Zadpoor 2017; Abbasi et al., 2020).

For the effects of the micropores on the biological responses in SBD reconstruction, the size of the micropores used in this study was less than 1 µm. Although micropores less than 1 µm did not directly promote bone ingrowth in the early stage, they promoted osteoclastogenesis followed by osteoclast–osteoblast interactions (Cappariello et al., 2014; Bohner et al., 2017; Hayashi et al., 2019a; Hayashi and Ishikawa 2020a; Kim et al., 2020). Osteoclastogenesis requires longer time than bone ingrowth into the scaffold *via* channels. This can be attributed to the micropore effects for SBD reconstruction in the middle stage.

Finally, we discuss the effects of the combined channels and micropores on the biological responses in SBD reconstruction. This study demonstrated that CAp HC scaffold with suitable volumes of channels and micropores exhibited superior osteoconductivity and scaffold replacement ability with new bones and bone marrow compared to scaffolds with suitable volume of either channels or micropores. In the case of cm-HC, most of the fibrous tissues within channels at the aperture region were replaced with new bones during 8 weeks. Meanwhile, in the case of c-HC and m-HC, fibrous tissues were still observed within some channels at the aperture region at 12 weeks postoperatively, suggesting that c-HC and m-HC did not achieve biomechanically stable states in SBDs owing to their low osteoconductivities, and bone remodeling did not start yet. Therefore, scaffolds with insufficient volume of channels or micropores are likely to delay the initiation of bone remodeling in SBDs. Zhang et al. (2017) reported the reconstruction of 10-mm-long SBDs in a rabbit radius using hollow-pipe-packed silicate and β-TCP scaffolds with channels having an aperture size of 500 µm. Although the new bone areas in these hollow-pipe-packed scaffolds at 12 weeks postoperatively were comparable to that in cm-HC in the present study, there were inadequate remnants of the hollow-pipe-packed scaffolds, and they were not replaced with the new bone (Zhang et al., 2017). This phenomenon likely occurred because of the overlooked importance of the micropores. Furthermore, the area of blood vessels in the CAp HC scaffold with suitable volumes of channels and micropores was the highest at 12 weeks postoperatively. Vascularization is a crucial finding because it can contribute to bone remodeling (Lafage-Proust et al., 2015). One of the reasons for the progression of bone remodeling in cm-HC was inferred to be the favorable vascularization in the scaffold. Although this study was unable to reveal whether channels or micropores had a greater impact on scaffold vascularization, the results in this study suggested that lack of volume for either channels or micropores might cause delayed vascularization in HC scaffolds, subsequently delaying bone remodeling. In detail, the above findings highlighted the roles of the channels and micropores, which indicated that the control of both channels and micropores is necessary to achieve scaffold replacement with new bones and bone marrow.

Although the present study did not focus on the channel shape, several researchers reported their effects on bone formation. Particularly, there have been several reports that a rectangular pore shape provides an intermediate effect on bone formation among various shapes, suggesting its unsuitability (VanBael et al., 2012; Entezari et al., 2019). VanBael et al. (2012) investigated the effect of three types of Ti6Al4V scaffolds with different pore shapes (triangular, rectangular, and hexagonal) on the proliferation and differentiation of human periosteum-derived cell cultures (hPDCs). They demonstrated the highest alkaline phosphatase (ALP) activity in the Ti6Al4V scaffolds with triangle pores, whereas those with rectangular and hexagonal pores were comparable. In addition, the amount of pore occlusion with hPDCs in the hexagonal pores was larger than that in other pore shapes, thereby decreasing the open space for cell distribution in hexagonal pores. Furthermore, Entezari et al. (2019) reported that strontium–Hardystonite–Gahnite scaffolds with quatrefoil-shaped channels provided a larger bone volume in the bone defect of rabbit calvaria and higher effective stiffness after implantation in the defect than those with rectangular channels. The abovementioned findings suggest that the bone formation ability of rectangular channels is presumed to be equal or superior to those of hexagonal channels, and inferior to those of triangle and quatrefoil-shaped channels.

The present study revealed that the mechanical strength of HC scaffolds depended on the micropore volume rather than the channel volume. Owing to the presence of micropores in the struts of HC scaffolds, the increase in the micropore volume decreased the strut robustness (Cordell et al., 2009; Ali et al., 2017). Furthermore, in HC scaffolds, the channels and struts were arranged parallel to one another; thereby, the struts were not separated by the channels and completely continuous. Owing to these reasons, even though the pore volume (∼0.3 cm^3^/g) and porosity (∼56%) in c-HC and m-HC were approximately equal, c-HC possessed higher mechanical strength than m-HC. Nevertheless, if the channels and struts were not arranged in parallel, the influence of the channels on the compressive strength was considerably high. In fact, even though the scaffold channels are unidirectional, the scaffold had a low compressive strength when the channels and struts were not parallel. For example, the compressive strengths of the unidirectional porous HAp and β-TCP scaffolds were 14 and 8 MPa, respectively, because the struts were discontinuous and separated (Makihara et al., 2018; Kumagai et al., 2019). Thus, owing to the HC structure, the HC scaffolds in this study minimally suppressed the strength reduction by increasing the channel volume. Consequently, the influence of the micropore volume was proportionally high.

Several researchers have attempted to reconstruct SBDs using MSCs or bone morphogenesis proteins (BMPs) in combination with scaffolds (Rathbone et al., 2014; Akilbekova et al., 2018). Rathbone et al. (2014) combined 3D porous hydroxyapatite scaffolds and MSCs for reconstructing 10-mm-long SBDs of the rabbit radius. Despite being combined with MSCs, the SBDs were not reconstructed, although the scaffolds with MSCs corresponded to a higher rate of bone healing compared to those without MSCs 2 weeks postoperatively. Akilbekova et al. (2018) used heparin-conjugated fibrin hydrogel scaffolds with BMPs for the reconstruction of 10-mm-long SBDs in the rabbit radius. The sole scaffold could not heal the defect. In contrast, the combined scaffolds and BMPs could connect the stumps of the host bone 12 weeks postoperatively. Thus, MSCs and BMPs have minimal and significant effect on bone regeneration, respectively. Notably, the use of MSCs and BMPs increases the treatment cost and involves concerns regarding safety, such as carcinogenesis and immune responses (Lukomska et al., 2019; Ramly et al., 2019). Furthermore, in existing studies on combined scaffolds and MSCs or BMPs, a cell-shielding membrane was not placed between the radius and the ulna (Rathbone et al., 2014; Akilbekova et al., 2018). In previous studies that achieved the formation of new bone connecting the stumps of the host bone in 10-mm-long SBDs, the efficacy of scaffolds was evaluated without using a cell-shielding membrane (Zhang et al., 2017; Chu et al., 2018; Tovar et al., 2018). In these cases, as new bone was formed from the radius (Bodde et al., 2008), new bone formation merely from the ulna might not be evaluated precisely. In this study, we placed the cell-shielding membrane to accurately evaluate the bone formation from the ulna, which rendered bone reconstruction more challenging than that without a cell-shielding membrane (Elbackly et al., 2015; Shibahara et al., 2021). Despite the more challenging evaluation environment, cm-HC in this study achieved the reconstruction of SBDs. Thus, the optimization of the channels and micropores can promote the efficacy of SBD reconstruction than the combined use of MSCs or BMPs. These findings can facilitate the development of scaffolds with a high ability of reconstructing SBDs without sacrificing cost and safety.

As a scaffold with similar HC structure, Osteopore® (Osteopore International Pte Ltd.), which is composed of polycaprolactone (PCL) and TCP (PCL:TCP = 80 wt%:20 wt%) and fabricated by three-dimensional printing, is well known (Rai et al., 2005, 2007; Bae et al., 2011; Lim et al., 2011; Holzapfel et al., 2019; Kobbe et al., 2020; Henkel et al., 2021). Researchers have attempted to reconstruct critical-sized SBDs using Osteopore® (Rai et al., 2005, 2007; Bae et al., 2011; Lim et al., 2011; Holzapfel et al., 2019; Kobbe et al., 2020; Henkel et al., 2021). Osteopore® combined with platelet-rich plasma (PRP) or BMPs or autografts successfully connected the stumps of the host bone with the new bone in animal studies and clinical trials (Rai et al., 2007; Bae et al., 2011; Lim et al., 2011; Holzapfel et al., 2019; Kobbe et al., 2020; Henkel et al., 2021). However, when Osteopore® alone was implanted in a critical-sized SBD, the stumps of the host were not fully connected during 12 weeks postoperatively (Rai et al., 2007; Bae et al., 2011). The results can be attributed to the absence of osteoconductivity of PCL, which was the main component of the composite HC scaffolds, resulting in the inflammatory response in the body (Xiao et al., 2012; Hasan et al., 2018). CAp has superior biocompatibility, osteoconductivity, and bioresorbability among several calcium phosphates (Ishikawa et al., 2018; Hayashi et al., 2019b, 2020b), and does not elicit any inflammatory response during resorption. Furthermore, Osteopore® was also burdened by the limitations of the mechanical strength. In detail, the compressive strength of Osteopore® was approximately 6.4 MPa (Rai et al., 2007; Bae et al., 2011; Lim et al., 2011), whereas that of CAp HC scaffolds in this study was at least 22 MPa. In general, the compressive strengths of the scaffolds fabricated by 3D printing are lower than those by extrusion molding (Hayashi et al., 2021c). Therefore, in terms of mechanical strength, CAp HC scaffolds are expected to be more advantageous than Osteopore®.

Although CAp HC scaffolds are promising for critical-size SBD reconstruction, there are several limitations in this study. First, this study did not evaluate the biomechanical strengths of the bones reconstructed by the CAp HC scaffolds. However, all CAp HC scaffolds used in this study did not crack until 12 weeks postoperatively, suggesting their sufficient biomechanical strengths required for SBD treatment. Second, the volume of bone newly formed in HC scaffolds was still low at 12 weeks postoperatively. However, the scaffolds occupied more than half of the defects in any observation period (Figure 6C). Consequently, the volume of tissues within the scaffold was less than half the volume of defects even if the scaffold was fully filled with regenerated tissues. In addition, the HC scaffolds did not fully cover the stumps of host bones (Supplementary Figure S5). In this environment, the average bone volume in the cm-HC was 10.6% at 12 weeks postoperatively. The volume percentage of the ulnar cortical bone in the diaphysis of the ulna is 35.2 ± 2.7% (Supplementary Figure S6). Therefore, cm-HC reconstructed approximately 30% of the ulna at 12 weeks postoperatively. We would like to clarify that HC scaffolds might be further replaced by bones in a long observation period in our future research. Third, the results of this study were obtained from experiments using small animals, i.e., rabbits, which may not perfectly correspond to the results obtained by preclinical experiments using large animals, such as sheep and pigs. As the bones of a large animal have higher similarity to human bones than those of small animals (Reichert et al., 2009; McGovern et al., 2018), experiments using large animals are preferred for preclinical experiments. Nevertheless, previous studies using Osteopore® demonstrated that the experimental results of small animal models can infer findings for large animal models (Rai et al., 2007; Bae et al., 2011; Lim et al., 2011; Henkel et al., 2021). Nonetheless, Osteopore® alone could not reconstruct critical-sized SBDs regardless of the animal size or species, i.e., rat, rabbit, or pig (Rai et al., 2007; Bae et al., 2011; Lim et al., 2011). To achieve reconstruction, the combination of Osteopore® and PRP or BMPs or autografts was necessary (Rai et al., 2007; Bae et al., 2011; Lim et al., 2011; Henkel et al., 2021). Although the results of experiments using small animals are not entirely consistent with those of experiments using large animals, they are useful for predicting the benefits of the scaffold. In our present study, CAp HC scaffolds with suitable volumes of the channels and micropores achieved the formation of new bone connecting the stumps of the host bone in critical-sized SBDs in the rabbit ulna. This result suggests the applicability of the CAp HC scaffolds for the reconstruction of critical-sized SBDs in large animals. To verify this inference, the efficacy of CAp HC scaffolds for the reconstruction of critical-sized SBDs in large animals will be evaluated in future research.

## Conclusion

In this study, three types of HC scaffolds were fabricated using extrusion molding. HC scaffolds with a larger volume of the channels than that of the micropores promoted bone ingrowth in the early stage (4 weeks postoperatively). Meanwhile, HC scaffolds with a larger volume of the micropores than that of the channels promoted scaffold resorption by osteoclasts, bone formation, and replacement of scaffolds with new bone in the medium term (12 weeks postoperatively). Thus, channels and micropores exerted different effects at different time points in SBD reconstruction. In addition, HC scaffolds with large volumes of both channels and micropores achieved replacement with new bone. The presented findings can help clarify the effect of channels and micropores, and their combination on SBD reconstruction, thereby facilitating the development of scaffolds with superior abilities.

## Data Availability

The original contributions presented in the study are included in the article/Supplementary Material, further inquiries can be directed to the corresponding author.
