# Supplementary material for: Effects of Channels and Micropores in Honeycomb Scaffolds on the Reconstruction of Segmental Bone Defects
Source: Front Bioeng Biotechnol. 2022 Mar 18;10:825831. doi: 10.3389/fbioe.2022.825831 (PMC8971796; doi:10.3389/fbioe.2022.825831)
Supplement: Supplementary file 1 [file DataSheet1.PDF]

Supporting Informations

# Effects of Channels and Micropores in Honeycomb Scaffolds on the Reconstruction of Segmental Bone Defects

**Keigo Shibahara[1,2], Koichiro Hayashi[1*], Yasuharu Nakashima[2], Kunio Ishikawa[1]**

[1]Department of Biomaterials Faculty of Dental Science, Kyushu University 3-1-1 Maidashi, Higashi-ku, Fukuoka 812–8582, Japan
[2]Department of Orthopedic Surgery, Graduate School of Medical Sciences, Kyushu University 3-1-1 Maidashi, Higashi-ku, Fukuoka 812–8582, Japan

*** Correspondence:**
Koichiro Hayashi
khayashi@dent.kyushu-u.ac.jp

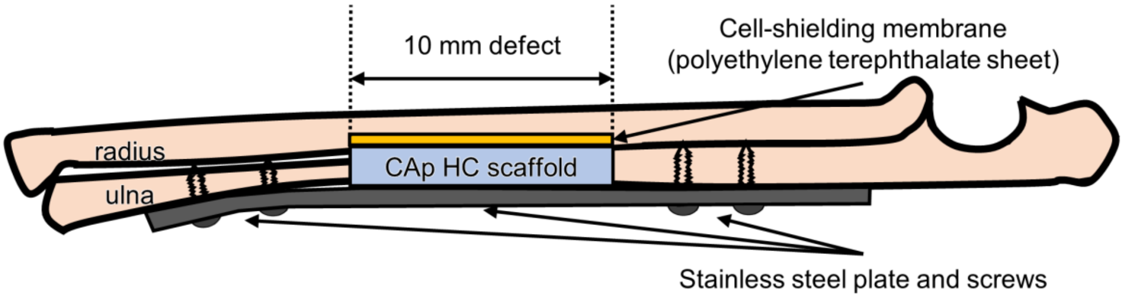


Figure S1. The illustration of the critical-sized animal model used in this study.

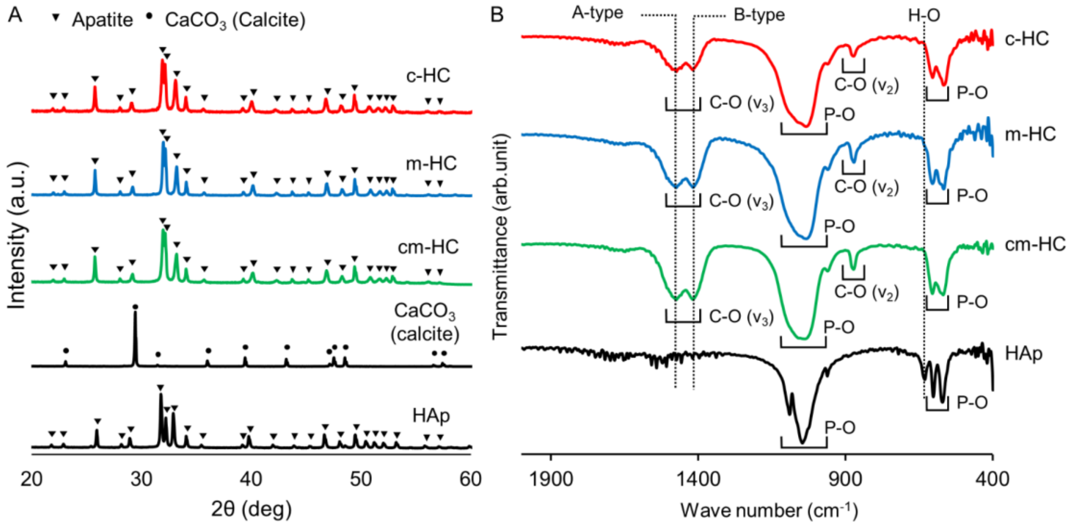


Figure S2. (A) XRD patterns of c-HC, m-HC, cm-HC, $CaCO_3$ (calcite), and HAp. (B) FTIR spectra of c-HC, m-HC, cm-HC, and HAp. (C–O), (P–O), and (H–O) indicate the carbonate, phosphate, and hydroxyl bands, respectively.

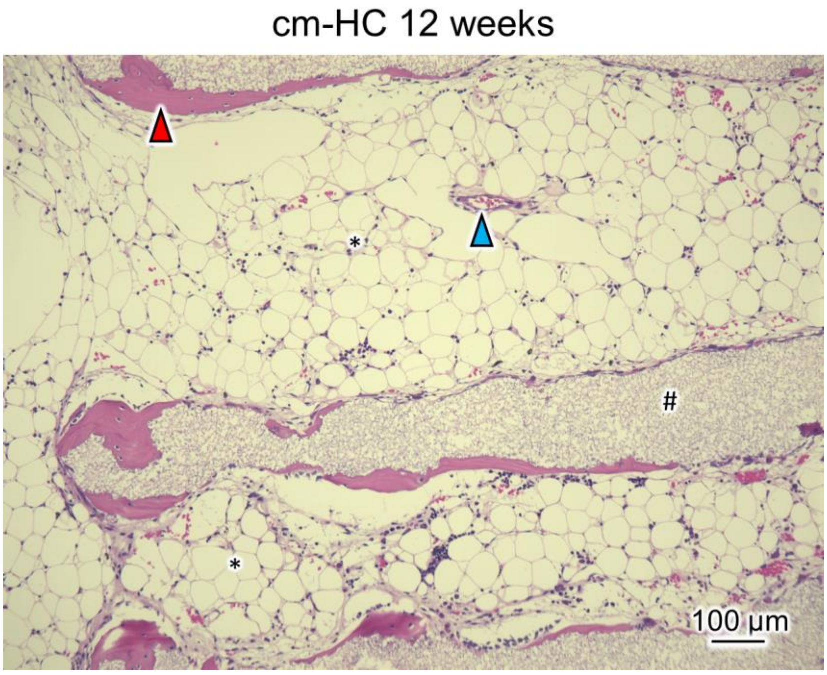


Figure S3. The histological images at channel aperture region corresponding to ulnar bone marrow. The red and blue arrowheads indicate mature bone and blood vessel, respectively; “#” and “*” indicate material and bone marrow, respectively. The left side of this image is a bone marrow region of ulna.

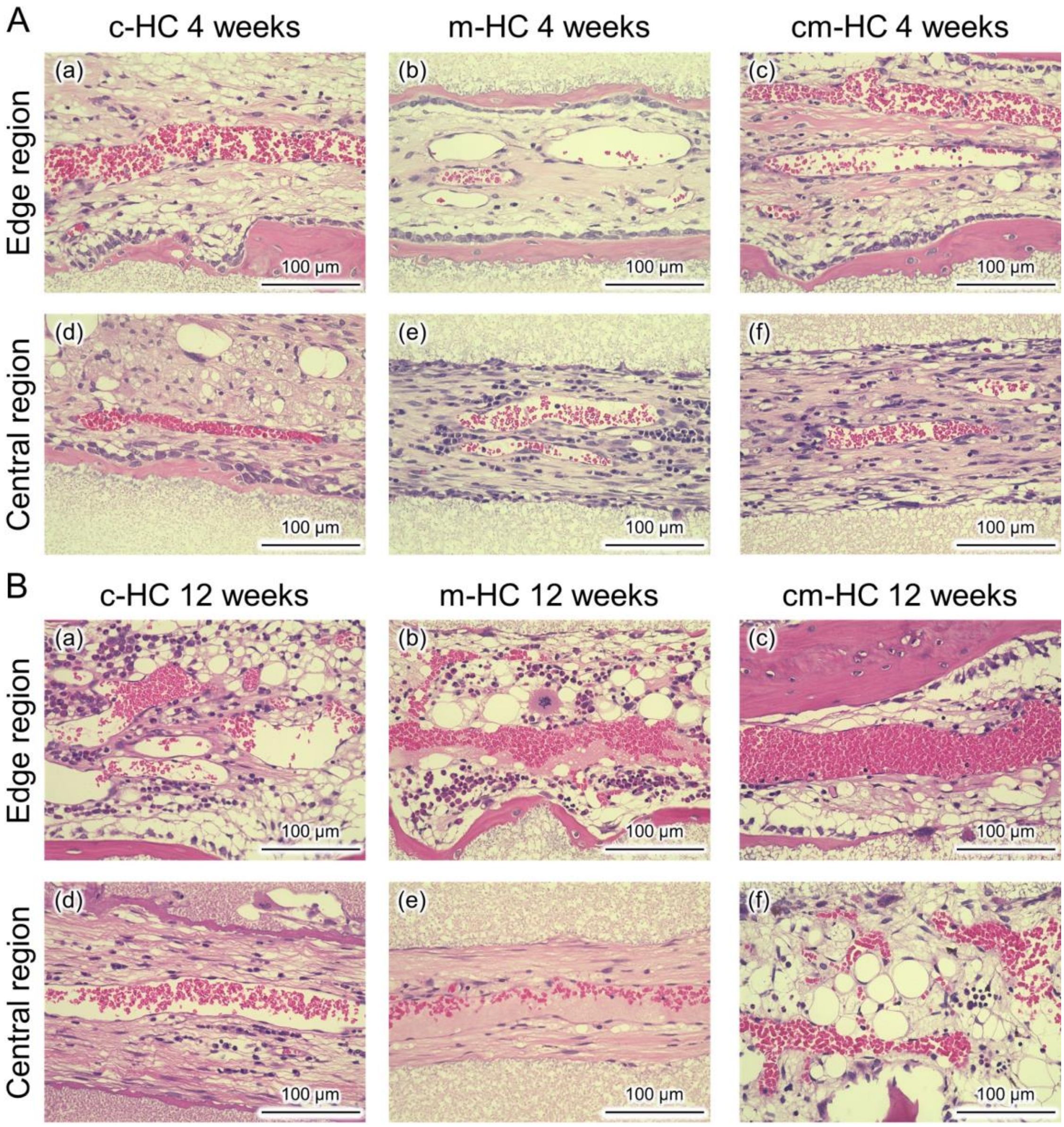


Figure S4. Typical HE-stained images of (a, d) c-HC, (b, e) m-HC, and (c, f) cm-HC at (A) four weeks and (B) 12 week postoperatively. Postoperatively, the images of (a), (b), and (c) show the edge region of HC scaffolds. The images of (d), (e), and (f) show the central region of HC scaffolds. The blue arrowhead indicates blood vessel.

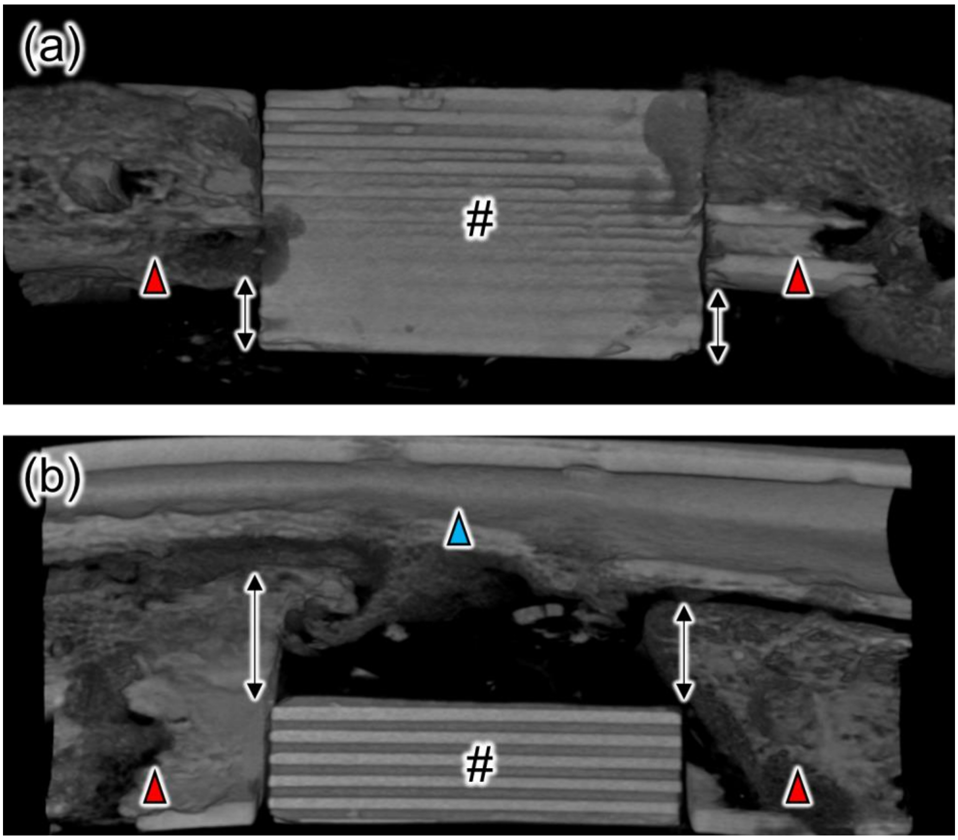


Figure S5. Typical µ-CT figures from (a, b) two directions showing that the HC scaffold incompletely covered the stump of host bone. The black arrow regions indicate the region without contact between HC scaffold and host bones. The red and blue arrowheads show ulna and radius, respectively; "#" indicates HC scaffold.

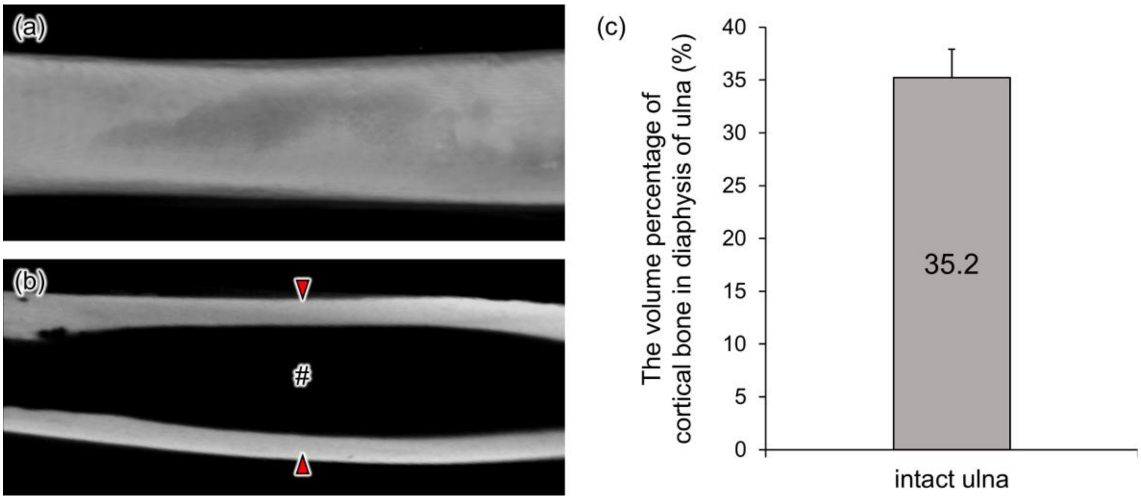


Figure S6. (a) Three-dimensional image of intact ulna. (b) Cross sectional image of intact ulna. The red arrowhead shows ulnar cortical bone; "#" indicates bone marrow region. (c) The volume percentage of cortical bone in diaphysis of ulna (%).
